# Nonalcoholic fatty liver disease and the risk of atrial fibrillation stratified by body mass index: a nationwide population-based study

**DOI:** 10.1038/s41598-021-83367-x

**Published:** 2021-02-12

**Authors:** So-Ryoung Lee, Kyung-Do Han, Eue-Keun Choi, Seil Oh, Gregory Y. H. Lip

**Affiliations:** 1 Department of Internal Medicine, Seoul National University Hospital, 101 Daehak-ro, Jongno-gu, Seoul, 03080 Republic of Korea; 2 Statistics and Actuarial Science, Soongsil University, Seoul, Republic of Korea; 3 Liverpool Centre for Cardiovascular Science, University of Liverpool and Liverpool Chest & Heart Hospital, Liverpool, UK; 4 Department of Clinical Medicine, Aalborg University, Aalborg, Denmark

**Keywords:** Cardiology, Risk factors

## Abstract

We evaluated the association between nonalcoholic fatty liver disease (NAFLD) and incident atrial fibrillation (AF) and analyzed the impact of NAFLD on AF risk in relation to body mass index (BMI). A total of 8,048,055 subjects without significant liver disease who were available fatty liver index (FLI) values were included. Subjects were categorized into 3 groups based on FLI: < 30, 30 to < 60, and ≥ 60. During a median 8-year of follow-up, 534,442 subjects were newly diagnosed as AF (8.27 per 1000 person-years). Higher FLI was associated with an increased risk of AF (hazard ratio [HR] 1.053, 95% confidence interval [CI] 1.046–1.060 in 30 ≤ FLI < 60, and HR 1.115, 95% CI 1.106–1.125 in FLI ≥ 60). In underweight subjects (BMI < 18.5 kg/m^2^), higher FLI raised the risk of AF (by 1.6-fold in 30 ≤ FLI < 60 and by twofold in FLI ≥ 60). In normal- and overweight subjects, higher FLI was associated with an increased risk of AF, but the HRs were attenuated. In obese subjects, higher FLI was not associated with higher risk of AF. NAFLD as assessed by FLI was independently associated with an increased risk of AF in nonobese subjects with BMI < 25 kg/m^2^. The impact of NAFLD on AF risk was accentuated in lean subjects with underweight.

## Introduction

Nonalcoholic fatty liver disease (NAFLD) is known as predominant macrovesicular steatosis of the liver^1^. NAFLD frequently coexists with comorbidities such as obesity, insulin resistance, hypertension, and dyslipidemia, which are risk factors for cardiovascular disease. Thus, NAFLD can be considered as the hepatic manifestation of metabolic syndrome^2^. The association between NAFLD and cardiovascular diseases has been reported for coronary artery disease, cardiomyopathy and cardiac arrhythmias^3–5^.

Atrial fibrillation (AF) is the most common cause of sustained cardiac arrhythmia, and the link between NAFLD and incident AF has been highlighted in recent years. Although several epidemiologic studies have suggested an association between NAFLD and the risk of AF, the results are somewhat conflicting, especially based on characteristics of the study population^6–9^.

Nonetheless, the prevalence of NAFLD has increased worldwide with the rising trend of sedentary lifestyle and the increasing rate of obesity. Currently, the global prevalence of NAFLD is reported to be more than 25%^2,10^. The prevalence of NAFLD in Asians is also increasing, and is reported to be 10 to 30%^10^. Despite the strong association between NAFLD and obesity, NAFLD is also seen in the nonobese population, and nonobese or lean NAFLD is more common in the Asian population^11–15^. There is limited data about the association between NAFLD and incident AF in the Asian population, who have a distinctively higher prevalence of nonobese NAFLD.

In this study, we evaluated the association between NAFLD as assessed by the fatty liver index (FLI) and incident AF in a large-scale observational cohort comprising the Asian population. In addition, we analyzed the impact of NAFLD on the risk of AF in relation to the body mass index (BMI).

## Methods

### Data sources

This study was based on the data from the Korean National Health Insurance Service (NHIS) database from 2009 to 2017. The Korean NHIS is a single insurer and a mandatory health insurance program that provides comprehensive medical care coverage for 97% of the Korean population managed by the Korean government. The Medical Aid program covers the remaining 3% of the population with low income. Information on Medical Aid beneficiaries was integrated into a single NHIS database since 2006. The NHIS database includes subjects’ demographics, utilization of inpatient and outpatient health care, pharmacy claims, data from health examination provided by the National Health Insurance Corporation, and mortality data for the entire Korean population^16^. Detailed information the participants of national health examination is presented in the Supplementary Methods. Diagnoses were coded using the *International Classification of Disease, Tenth, Clinical Modification* (ICD-10-CM) codes. This study complied with the Declaration of Helsinki. This study was exempt from review by the institutional review board of Seoul National University Hospital (E-1612-036-812).

### Study population

**Figure 1:**
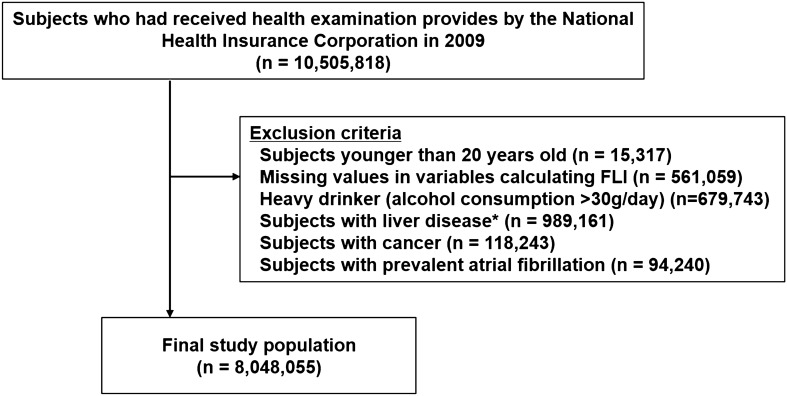
Study enrollment flow. *Liver disease: obvious liver disease such as liver cirrhosis (ICD-10 codes, K703 and K76) and hepatitis (ICD-10 codes, B15–B19), (v) existing cancer (ICD-10 codes, C00-96).

This study included the subjects who underwent the national health examination in 2009 (n = 10,505,818). The exclusion criteria were as follows; (i) subjects aged under 20 years, (ii) subjects with missing health examination data, (iii) subjects with excessive alcohol intake (> 30 g/day), (iv) patients with obvious liver disease such as liver cirrhosis (ICD-10-CM codes, K703 and K76) and hepatitis (ICD-10-CM codes, B15-B19), (v) patients with prevalent cancer (ICD-10-CM codes, C00-96), and (vi) patients with prevalent AF (ICD-10-CM codes, I480-I484, I489) before 2009 (Fig. 1). Finally, a total of 8,048,055 subjects was included in the analysis (Fig. 1).

### Definition of nonalcoholic fatty liver disease

In the previous studies, NAFLD was diagnosed using various methods such as ultrasonography, computed tomography, FLI, or circulating levels of liver enzymes^11–15,17–21^. According to the definition of NAFLD, evidence of hepatic steatosis either on imaging or from the histology is mandatory^1^. However, these methods involve high costs and are not usually feasible for the screening of fatty liver in a large population-based cohort. FLI is one of the alternative methods to diagnose NAFLD, and is a validated score using BMI, waist circumference, serum levels of γ-glutamyl transferase (GGT) and triglyceride^22,23^. FLI < 30 rules out, while FLI ≥ 60 indicates fatty liver disease with good diagnostic accuracy^22,23^. Therefore, we categorized subjects into 3 groups according to FLI as follows: FLI < 30, 30 ≤ FLI < 60, and FLI ≥ 60.

### Covariates

The subjects’ demographic information including age, sex, socioeconomic status, comorbidities, the results of general health examination were collected. Comorbidities including hypertension, diabetes mellitus, and dyslipidemia were identified from the medical claim records with relevant ICD-10-CM codes and prescription records (Supplementary Table S1). Low income was defined as the lowest income at 25% among the entire Korean population. The general health examination included height, weight, waist circumference, systolic and diastolic blood pressure, and laboratory tests such as fasting glucose, total cholesterol, triglyceride, serum creatinine and liver enzymes (alanine transaminase [ALT], aspartate transaminase [AST], and GGT). Chronic kidney disease was defined as an estimated glomerular filtration rate of ≤ 60 mL/min/1.73 m^2^ calculated using the Modification of Diet in Renal Disease formula. Using the standardized self-reporting questionnaires, information on smoking (never, ex-, and current smoker), alcohol consumption (non-drinker, and mild to moderate alcohol consumption defined as alcohol intake 0 to 30 g/day), and regular exercise were obtained^16^. Regular exercise was defined as performing longer than 30 min of moderate physical activity 5 or more times per week or longer than 20 min of strenuous physical activity 3 or more times per week. BMI was calculated as weight in kilograms divided by the square of height in meters (kg/m^2^). BMI was classified into 5 categories following World Health Organization recommendation for Asians: underweight, < 18.5 kg/m^2^; normal weight, 18.5–22.9 kg/m^2^; overweight, 23.0–24.9 kg/m^2^; obese class I, 25.0–29.9 kg/m^2^; and obese class II, ≥ 30.0 kg/m^2^^24,25^.

### Study outcome and follow-up

The main study outcome was new-onset AF during follow-up. New-onset AF was defined as at least 1 hospital visit with a diagnosis of AF according to the ICD-10-CM codes (I480-I484, I489) since the time of enrollment into the study^26^. The cohort was followed up until the occurrence of incident AF, disqualification from the NHIS (death or immigration), or end of the study period (December 31, 2017), whichever came first.

### Statistical analysis

Baseline characteristics were described using percentage for categorical variables and mean ± standard deviation or median (interquartile ranges) for continuous variables. One-way analysis of variance for continuous variables and the chi-square test for categorical variables were used to evaluate the significance of differences among the groups stratified by FLI. The crude incidence rate (IR) of incident AF was calculated by dividing the number of AF cases by the total follow-up period (per 1000 person-years). Cox proportional hazard regression models were used to evaluate the association between each variable and the occurrence of new-onset AF. Hazard ratios (HRs) and 95% confidence intervals (CIs) were calculated.

Multivariable regression models were created and adjusted for age and sex (model 1); age, sex, hypertension, diabetes mellitus, dyslipidemia, chronic kidney disease, smoking status, alcohol consumption, regular exercise, and low income (model 2); model 3 was adjusted for the variables listed above, as well as for systolic blood pressure, fasting glucose, and total cholesterol. HRs for AF incidence were estimated in subjects with 30 ≤ FLI < 60 and FLI ≥ 60 and compared with subjects who had FLI < 30 as a reference group. Covariates for models 1 and 2 were selected based on a priori knowledge of risk factors for AF, and model 3 included all variables in model 2 plus the variables included in health examinations which have been well-established as risk factors of incident AF^27,28^. The proportional hazards assumption for Cox models was graphically tested with a log minus log graph and verified with the Schoenfeld residuals. The results showed parallel log minus log survival curves and random patterns in Schoenfeld residuals, indicating no significant departure from proportionality assumption, and the tests were not statistically significant. Multi-collinearity was evaluated using the variance inflation factor (VIF). There was no substantial collinearity between the covariates (VIF = 1.38–1.78). To avoid the possible effect of multi-collinearity caused by correlation among BMI, waist circumference, GGT, and triglyceride, we did not include these variables in the multivariable Cox model.

We hypothesized that FLI might be a useful marker to estimate the metabolic status and the risk of AF in lean Asian subjects. To evaluate the impact of FLI on the risk of AF in different BMI groups, we performed a subgroup analysis in different BMI groups as an exploratory analysis. In this analysis, we performed multivariable Cox analysis using model 3, including age, sex, hypertension, diabetes, dyslipidemia, chronic kidney disease, smoking, alcohol consumption, exercise, low income, systolic blood pressure, total cholesterol, and fasting glucose. Interactions between FLI and BMI relating to incident AF were calculated. Cumulative incidences of AF in the FLI groups within the different BMI groups were plotted using Kaplan–Meier analyses, and the long-rank test was performed to evaluate the differences between the groups.

p values < 0.05 were considered statistically significant. The Bonferroni significance was reported for multiple comparisons. A Bonferroni-correction significance level of p < 0.004 was used for 12 multiple tests (two FLI comparisons in all subjects and in each of five BMI categories) in model 3. Statistical analyses were performed using SAS version 9.4 (SAS Institute, Cary, North Carolina).

## Results

### Baseline characteristics

The mean FLI of the total study population was 26.0 ± 23.3. Among a total of 8,048,055 subjects, 22.5% (n = 1,814,124) were categorized into the 30 ≤ FLI < 60 group, and 11.5% (n = 924,497) into the FLI ≥ 60 group. Baseline characteristics according to FLI ranges are presented in Table 1. A higher percentage of subjects with higher FLI value were men, and had comorbidities such as hypertension, diabetes mellitus, and dyslipidemia. In addition, subjects with higher FLI value had higher BMI, waist circumference, systolic and diastolic pressure, fasting glucose, total cholesterol, triglyceride, AST, ALT, and GGT, but lower HDL-C. A higher percentage of subjects with higher FLI value were current smokers, mild alcohol consumers and undertook regular exercise; however, there was a lesser percentage of subjects with low income.

**Table 1:** Baseline characteristics of study subjects stratified by fatty liver index.

	Total (n = 8,048,055)	Fatty liver index group		
		0–30 (n = 5,309,434)	30–60 (n = 1,814,124)	≥ 60 (n = 924,497)
Age	46.7 ± 14.1	45.4 ± 14.4	50.0 ± 13.5	47.3 ± 12.7
Men	52.0	40.9	69.5	81.6
Height (cm)	163.6 ± 9.2	162.3 ± 9.0	165.1 ± 9.4	167.6 ± 8.7
Weight (kg)	63.4 ± 11.5	58.6 ± 8.7	69.7 ± 8.8	78.6 ± 10.8
BMI (kg/m^2^)	23.6 ± 3.2	22.2 ± 2.4	25.5 ± 2.2	27.9 ± 3.0
Waist circumference (cm)	79.8 ± 9.1	75.5 ± 7.0	85.9 ± 5.4	92.1 ± 6.8
Systolic BP (mmHg)	122.0 ± 14.9	119.1 ± 14.3	126.5 ± 14.3	129.9 ± 14.5
Diastolic BP (mmHg)	76.0 ± 9.9	74.1 ± 9.5	78.8 ± 9.5	81.5 ± 9.9
Fasting glucose (mg/dL)	96.5 ± 22.1	93.2 ± 17.9	100.7 ± 25.1	106.7 ± 31.4
Total cholesterol (mg/dL)	195.2 ± 36.5	189.2 ± 34.4	204.0 ± 36.6	212.7 ± 38.8
Triglyceride (mg/dL)	108 (74–160)	87 (64–118)	154 (117–204)	226 (165–313)
GGT (mg/dL)	23 (16–37)	19 (14–25)	35 (25–50)	61 (40–97)
AST (mg/dL)	23 (19–28)	22 (19–26)	25 (21–30)	30 (24–38)
ALT (mg/dL)	20 (15–29)	18 (14–23)	26 (20–36)	37 (27–53)
Hypertension	24.1	17.1	34.9	43.3
Diabetes mellitus	7.9	4.7	12.0	17.8
Dyslipidemia	17.6	12.1	25.2	33.9
CKD (eGFR ≤ 60 mL/min/1.73 m^2^)	5.8	5.3	6.9	6.3
**Smoking status**				
Non-smoker	62.3	70.5	50.2	38.6
Ex-smoker	13.3	10.3	18.6	19.9
Current smoker	24.5	19.2	31.2	41.6
**Alcohol consumption**				
Non-drinker	54.2	59.0	48.6	37.4
Mild (0–30 g/day)	45.8	41.0	51.4	62.6
Regular exercise	51.0	49.9	52.6	54.2
Low income	27.0	28.7	24.1	23.4

Categorical variables are presented as percentage and continuous variables are presented as mean ± standard deviation or median (interquartile ranges).

*ALT* alanine transaminase, *AST* aspartate transaminase, *BMI* body mass index, *BP* blood pressure, *CKD* chronic kidney disease, *eGFR* estimated glomerular filtration rate, *GGT* gamma-glutamyl transferase, *HDL* high density lipoprotein.

### FLI and the risk of incident AF

During a median follow-up period of 8.3 years (interquartile ranges [IQR] 8.1–8.6 years), 534,442 subjects were newly diagnosed with AF (IR, 8.27 per 1000 person-years). Supplementary Table S2 shows unadjusted HRs for incident AF for all independent variables. Higher age, female sex, as well as higher BMI, waist circumference, systolic and diastolic blood pressure, fasting glucose, total cholesterol, AST, ALT, GGT were associated with increased risk of incident AF. Hypertension, diabetes mellitus, dyslipidemia, and chronic kidney disease were also associated with an increased risk of AF.

The crude IRs of AF were 7.49, 9.95, and 9.48 per 1000 person-years in subjects with FLI < 30, 30 ≤ FLI < 60, and FLI ≥ 60, respectively (Table 2). On univariable analysis, HR of FLI as a continuous variable per units of 10 for the risk of AF was 1.061 (95% CI 1.060–1.062). Compared to subjects with FLI < 30, those with 30 ≤ FLI < 60, and FLI ≥ 60 showed a higher risk of incident AF (HR 1.328, 95% CI 1.320–1.337 and HR 1.267, 95% CI 1.257–1.278, respectively).

**Table 2:** Hazard ratios of the fatty liver index for incident atrial fibrillation.

FLI group	Total number	AF cases	IR*	Model 1	Model 2	Model 3
0–30	5,309,434	320,433	7.49	1 (reference)	1 (reference)	1 (reference)
30–60	1,814,124	144,052	9.95	1.097 (1.090–1.104)	1.038 (1.031–1.045)	1.053 (1.046–1.060)^a^
≥ 60	924,497	69,957	9.48	1.188 (1.178–1.198)	1.090 (1.080–1.100)	1.115 (1.106–1.125)^a^

Model 1: age sex.

Model 2: Model 1 + hypertension, diabetes, dyslipidemia, chronic kidney disease, smoking, alcohol consumption, exercise, low income.

Model 3: Model 2 + systolic blood pressure, total cholesterol, fasting glucose.

*AF* atrial fibrillation, *FLI* fatty liver index, *IR* incidence rate.

*IR, incidence rate (per 1000 person-years).

^a^Bonferroni-corrected significance level of p < 0.004 was used for multiple comparisons.

After adjustment for age and sex, subjects with 30 ≤ FLI < 60 and FLI ≥ 60 showed a higher risk of incident AF (HR 1.097, 95% CI 1.090–1.104 and HR 1.188, 95% CI 1.178–1.198, respectively) than those with FLI < 30 (Supplementary Table S2). The increased risk in subjects with 30 ≤ FLI < 60 and FLI ≥ 60 was 10% and 19%, respectively. Even when adjusted for the clinical variables in model 3, subjects with 30 ≤ FLI < 60 and FLI ≥ 60 were independently and significantly associated with increased risks of AF by 5% and 12%, respectively (Table 2).

### Exploratory subgroup analysis: FLI and the risk of incident AF according to different BMI ranges

In the total study population, 4% (n = 323,667) were classified as underweight, 40.5% (n = 3,257,976) as normal weight, 24.6% (n = 1,977,281) as overweight, 27.6% (n = 2,223,616) as obese class I, and 3.3% (n = 265,515) as obese class II. After dividing the subjects into 5 different BMI groups, In nonobese subjects (underweight, normal weight, and overweight groups, all groups with BMI < 25 kg/m^2^), crude IRs increased with increasing FLI, and this dose–response relationship was more significant in underweight subjects (Supplementary Table S3). Obese subjects (BMI ≥ 25 kg/m^2^) did not show an association between FLI and the risk of AF.

**Figure 2:**
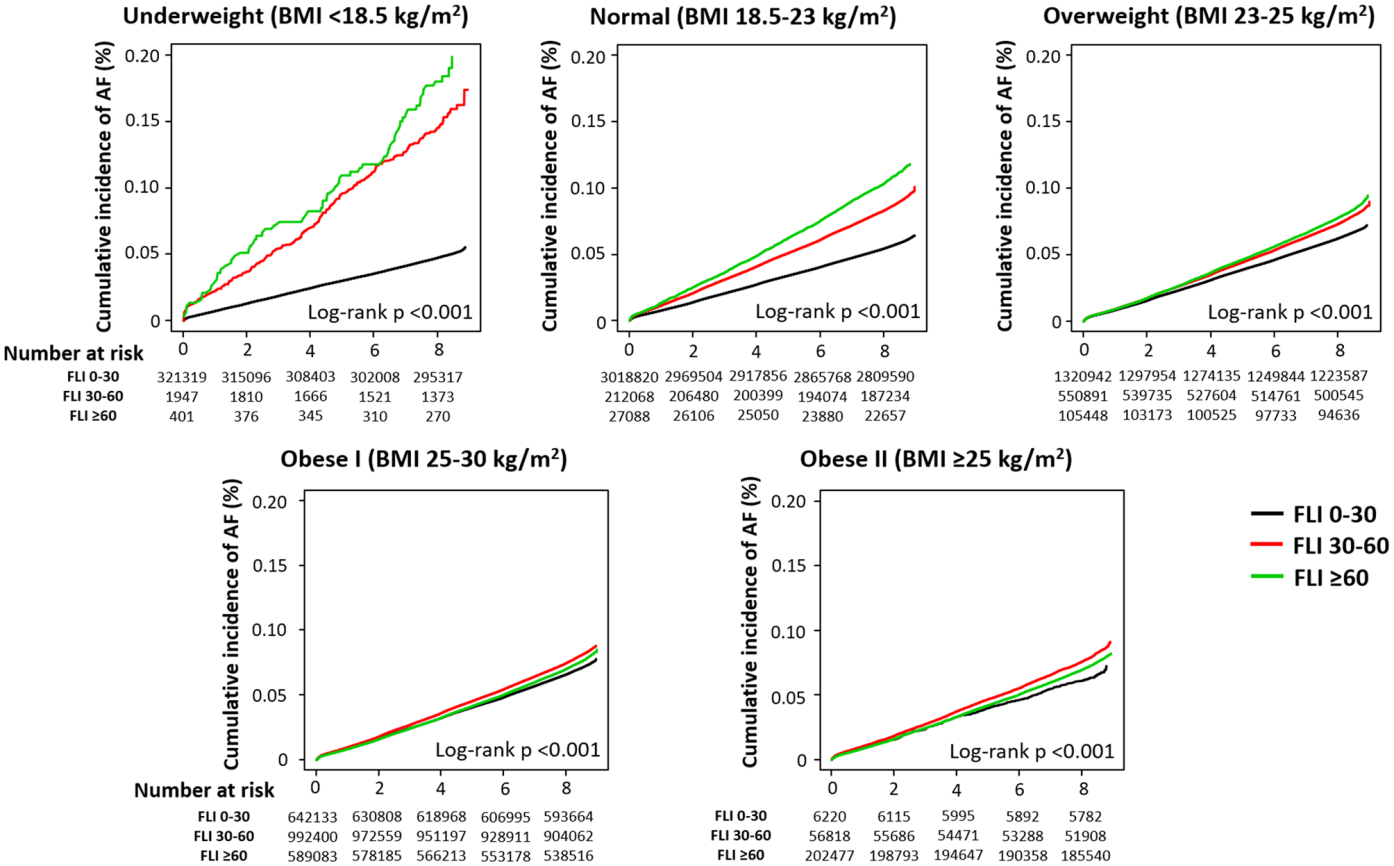
Cumulative incidence curves of AF stratified by fatty liver index in different BMI groups: underweight (BMI < 18.5 kg/m^2^), normal weight (BMI 18.5–23 kg/m^2^), overweight (BMI 23–25 kg/m^2^), obese I (BMI 25–30 kg/m^2^), and obese II (BMI ≥ 30 kg/m^2^). *AF* atrial fibrillation, *BMI* body mass index, *FLI* fatty liver index.

**Figure 3:**
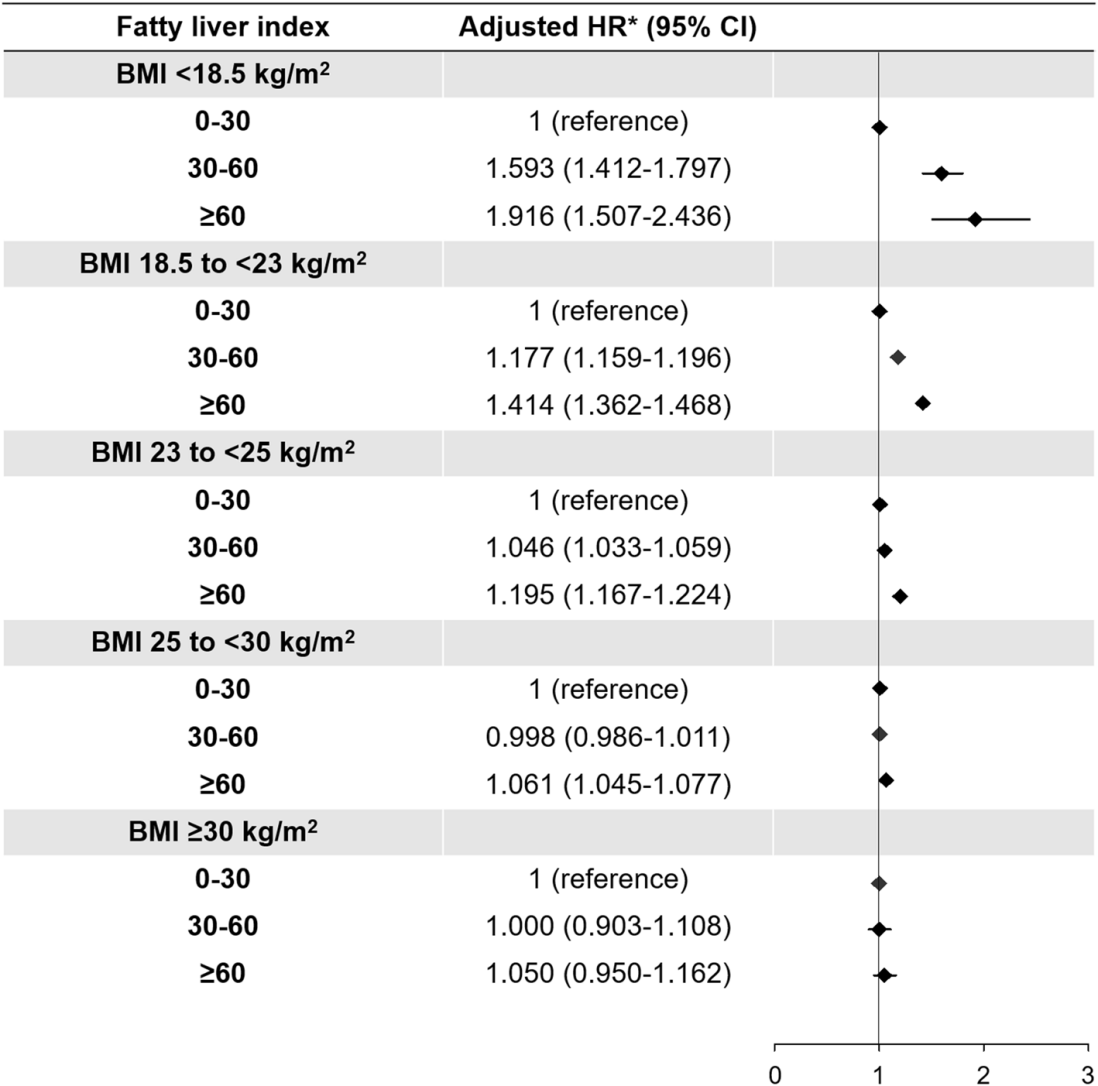
Hazard ratios of the FLI for incident AF in different BMI groups. *Adjusted HR: Model 3 (age, sex, hypertension, diabetes, dyslipidemia, chronic kidney disease, smoking, alcohol consumption, exercise, low income, systolic blood pressure, total cholesterol, and fasting glucose). *AF* atrial fibrillation, *BMI* body mass index, *CI* confidence interval, *FLI* fatty liver index, *HR* hazard ratio.

Cumulative crude incidence of AF was significantly associated with increasing FLI in underweight, normal weight, and overweight groups (Fig. 2). Although log-rank p values were also significant in obese class I and II groups due to the large sample size, the incidence of AF was less impacted by the increase in FLI and a linear dose–response relationship was not observed. After adjustment for the clinical variables (model 3), we found that high FLI (30 ≤ FLI < 60 and FLI ≥ 60) dramatically increased the risk of incident AF in underweight subjects (Fig. 3, Supplementary Table S3). In underweight subjects, FLI between 30 and 60 was associated with a 1.6 times increase in incident AF while FLI ≥ 60 increased the risk of AF risk by 2 times compared with that of FLI < 30. In normal weight and overweight subjects, high FLI was also significantly associated with increased risk of incident AF, but the HRs were attenuated. For subjects with obesity class I and II, high FLI did not have any significant effect on the risk of incident AF, except for a modest effect of FLI ≥ 60 in obese class I subjects (Fig. 3, Supplementary Table S3).

## Discussion

To our knowledge, this is the largest cohort study examining the association between NAFLD assessed by FLI and incident AF, especially in the Asian population. We have demonstrated the following principal outcomes: (i) among the entire Korean adult population who underwent health examination provided by the government in 2009, NAFLD was common; 22.5% were categorized as 30 ≤ FL I < 60 and 11.5% had FLI ≥ 60; (ii) subjects with 30 ≤ FLI < 60 and FLI ≥ 60 were associated with an increased risk of AF by 5% and 12%, respectively; (iii) when stratified according to BMI, high FLI (30 ≤ FLI < 60 and FLI ≥ 60) dramatically increased the risk of incident AF, especially in underweight subjects; however, this was attenuated in normal weight and overweight subjects; and (iv) in subjects with obesity class I and II, high FLI did not have any significant association with the risk of incident AF, except for a modest effect of FLI ≥ 60 in obese class I subjects.

AF is the most common cause of sustained cardiac arrhythmia, and is associated with increased risks of stroke, heart failure, and mortality^29^. The association between the incidence of AF and metabolic syndrome is well known^30,31^. NAFLD frequently coexists with the components of metabolic syndrome, hence the impact of NAFLD on the risk AF risk has drawn attention.

However, only limited data related to NAFLD and the risk of AF has been published, with conflicting results^6–9^. A recent meta-analysis analyzed 5 previous studies reporting the association between NAFLD and prevalence of AF^9^. NAFLD was diagnosed using various modalities like ultrasonography, computed tomography, or diagnostic codes. In a pooled analysis, NAFLD was associated with a higher prevalence of AF (odds ratio 2.07, 95% CI 1.38–3.10); however, there were only 3 original papers evaluating the association between NAFLD and incidence of AF in longitudinal cohorts^6–8^.

Targher et al. included subjects with type 2 diabetes (Italian; total 400 subjects, 281 subjects with NAFLD assessed by ultrasonography)^6^, and 2 other studies included middle-aged subjects; from Finland (total 958 subjects, 249 subjects with NAFLD assessed by ultrasonography)^7^, and from United States (total 2060 subjects, 424 subjects with NAFLD assessed by computed tomography)^8^. In a pooled analysis, NAFLD was not significantly associated with an increased risk of incident AF in the overall study population (HR 1.34, 95% CI 0.92–1.95)^9^. In fact, NAFLD had an increased risk of incident AF only in diabetic patients (HR 4.96, 1.42–17.28)^6,9^. Although NAFLD was diagnosed using different imaging modalities in these previous studies, there might be some limitations when comparing these results with our present study. Of note, we found no comprehensive large population-based longitudinal cohort data from these previous studies. Notably, there were no data about Asian subjects.

Furthermore, despite NAFLD commonly coexisting with obesity, nonobese NAFLD is common in Asians^11–15^. Although FLI has no or only a modest impact on the risk of incident AF in the general population, we hypothesized that there might be a significant interaction between the effect of FLI on the risk of AF, based on different BMI categories^32^. We found that subjects with 30 ≤ FLI < 60 and FLI ≥ 60 were associated with increased risks of AF by 5% and 12% respectively, in the overall study population. However, we have demonstrated for the first time a significant association between BMI and the impact of FLI on incident AF. In subjects with lower BMI, high FLI was more strongly associated with an increased risk of incident AF. Compared to the normal weight with normal FLI group, underweight subjects with FLI ≥ 60 showed a 2.1 times increase in the risk of incident AF (Supplementary Table S4). Obese subjects showed an increased risk of incident AF compared to the normal weight with normal FLI group, and FLI did not have an additional impact on the risk of incident AF among obese subjects (Supplementary Table S4).

NAFLD could be found in nonobese (BMI < 25 kg/m^2^) and lean (BMI < 23 kg/m^2^) subjects; more importantly, NAFLD appears to be more frequent in the Asian population^11–15^. The histology of nonobese NAFLD does not differ from that of obese NAFLD. In a recent report, lean NAFLD had a lower degree of steatosis and less advanced fibrosis but more severe lobular inflammation compared to obese NAFLD^33^. Indeed, nonobese NAFLD is a similar spectrum to the “metabolically obese normal weight (MONW)” that shows insulin resistance with normal weight.

BMI represents general obesity but sometimes fails to reflect body fat distribution. In contrast, visceral abdominal adiposity is associated with metabolic syndrome and is a key link to NAFLD^15^. Of note, Asian nonobese NAFLD patients have a higher body fat content and evidence of visceral adiposity than subjects with a comparable BMI without NAFLD^11^. Furthermore, the association with metabolic syndrome components was stronger in nonobese NAFLD than in obese NAFLD^34^.

In the present study, when dividing the subjects according to the BMI, the associations between FLI and the risk of AF was accentuated in nonobese groups (underweight and normal BMI groups). We can assume that triglyceride and GGT among four components of FLI might be the main parameters reflecting subjects’ metabolic healthiness in nonobese subjects. Triglyceride is one of the components to diagnose the metabolic syndrome^35^, and GGT is a well-known parameter that is independently associated with the development of obesity, hypertension and insulin resistance, which are components of metabolic syndrome^36–38^. In a previous study, we found a close association between GGT and the AF risk; although the obese group generally showed higher AF incidence than the nonobese group, the association between GGT and the AF risk was more accentuated in the nonobese population^32^. Nonobese NAFLD subjects generally have a higher prevalence of the components of metabolic syndrome, including high TG, high blood pressure, insulin resistance such as impaired fasting glucose, and overt diabetes^34,39^. Thus, it would be important to identify high-risk nonobese NAFLD patients and correct their metabolic profile by reducing visceral fat through dietary changes and regular physical activity.

There is also a paucity of data about the long-term prognosis of nonobese NAFLD patients. In a recent study, lean NAFLD subjects had a higher overall and cardiovascular mortality than the obese NAFLD subjects, but such data are still controversial^33,40^. Our study clearly shows an increased risk of incident AF in nonobese and lean NAFLD patients. Further studies on cardiovascular clinical outcomes in nonobese NAFLD subjects are warranted.

### Study limitations

Several limitations should be considered in this study. First, although we used the Korean NHIS database, which included the entire Korean population and a large number of patients received the national health examinations (66.0% in 2009)^41^, there is still a possibility of selection bias that the present analysis was performed in subjects who actually underwent the national health examination. Second, incident AF was identified using diagnostic codes in the claims database, which might result in misclassification due to both, underestimation and overestimation of AF. To avoid misclassification, we used the validated definitions from a previous study using the Korean NHIS database^26^. However, there is still a possibility that undetected AF patients with asymptomatic AF who were not treated by any medical service could exist. Third, using FLI as a surrogate measure for NAFLD could be a limitation of our study. In this large population-based study, the results of ultrasound, computed tomography, magnetic resonance imaging, or liver biopsy were not available to evaluate NAFLD. In addition, the diagnostic accuracy of each study is different, and the diagnostic ability of these tools varies depending on the sample population^2^. For example, Bedogni et al. developed FLI based on the diagnosis of the fatty liver using ultrasound, and FLI provided good diagnostic accuracy for fatty liver in the original study population (area under the receiver operating characteristics [AUROC] = 0.85, 95% CI 0.81–0.88)^22^. The characteristics of the study population in the previous study (mean age 57 years, 61.5% men, and mean BMI 27 kg/m^2^) were slightly different from those of our study population. Nonetheless, external validation of FLI in other populations showed comparable diagnostic accuracy for NAFLD^42^. Koehler et al.^42^ reported the external validation of FLI collecting ultrasonography in the Rotterdam Study (2652 subjects). NAFLD was present in 925 (34.9%) of participants, and the median FLI was 42.2 (interquartile ranges 22.6–67.3). Applying cutoffs proposed by Bedogni et al.^22^, 926 participants (34.9%) had FLI < 30, and 864 participants (32.6%) had FLI ≥ 60. AUROC of FLI for predicting NAFLD was 0.813 (95% CI 0.797–0.830). Sensitivity and specificity of FLI < 30 for predicting the absence of NAFLD were 91.5% and 49.0%, respectively. The sensitivity and specificity of FLI ≥ 60 for predicting the presence of NAFLD were 60.4% and 92.3%, respectively. Also, several previous studies tried to validate the FLI using ultrasonography, including in the Asian population^43–47^. Given that the results of imaging studies for diagnosing NAFLD could not be obtained in the present study, we could not perform further validation of FLI for diagnosing NAFLD in our dataset, which remains our major limitation. Fourth, although we assessed all the available covariates and included a multivariable analysis for adjusting the potential confounding factors of incident AF, some unmeasurable residual confounding factors that might be not accessible in the NHIS database could exist. Moreover, we did not assess longitudinal changes in the metabolic status of either FLI or its components (such as waist circumference, BMI, triglyceride and GGT levels or treatment) on NAFLD or other chronic diseases during the follow-up period. These might be confounding factors that could affect the incidence of AF. Fifth, variables such as smoking status, alcohol consumption, and exercise were assessed by self-reported questionnaires, including the national health examination. Although these variables from the Korean NHIS database have been widely used in many previous studies^48–50^, there might be a possibility of inaccuracy or recall bias in this information. Lastly, since this is a retrospective observational cohort study, our findings suggest an association between the presence of NAFLD as assessed by FLI with incident AF, but this does not indicate a causal relationship.

## Conclusions

In this large-scale Asian population, NAFLD assessed by FLI was independently associated with a higher risk of incident AF. The impact of NAFLD on AF risk might be accentuated in lean subjects with low weight (BMI < 18.5 kg/m^2^) and in those with normal weight (BMI 18.5–22.9 kg/m^2^).

## Supplementary Information

Supplementary Information.
