# Supplementary material for: Nonalcoholic fatty liver disease and the risk of atrial fibrillation stratified by body mass index: a nationwide population-based study
Source: Sci Rep. 2021 Feb 12;11:3737. doi: 10.1038/s41598-021-83367-x (PMC7881181; doi:10.1038/s41598-021-83367-x)
Supplement: Supplementary file 1 — Supplementary Information. [file 41598_2021_83367_MOESM1_ESM.docx]

**Nonalcoholic fatty liver disease and the risk of atrial fibrillation stratified by body mass index**

So-Ryoung Lee, MD,^a^* Kyung-Do Han, PhD,^b^* Eue-Keun Choi, MD, PhD,^a^ Seil Oh, MD, PhD^a^, Gregory Y. H. Lip, MD^a,c,d^

^a^ Department of Internal Medicine, Seoul National University Hospital, Seoul, Republic of Korea

^b^ Statistics and Actuarial Science, Soongsil University, Seoul, Republic of Korea

^c^ Liverpool Centre for Cardiovascular Science, University of Liverpool and Liverpool Chest & Heart Hospital, Liverpool, United Kingdom; and ^d^ Department of Clinical Medicine, Aalborg University, Aalborg, Denmark

*Two authors contributed equally.

**Supplementary Materials**

**Supplementary Methods**

**Supplementary Tables**

**Supplementary Methods**

*The national health examination provided by the Korean NHIC*

The purpose of the national health examination (named as the national health screening program) is the health promotion and screening of chronic diseases such as diabetes mellitus, hypertension, dyslipidemia, and other laboratory abnormalities (kidney/liver function and chest X-ray). In our study inclusion period (from January 1, 2009 to December 31, 2009), the eligibility of general health examination was as follows: (i) the employed and the self-employed who are the householders of a family, biannually; (ii) dependents of the employed and family members of the self-employed household aged 40 years or older, biannually; and (iii) medical aid beneficiaries, biannual (householders 19-64 years of age and family members 41-64 years of age) [1].

Reference

[1] Cheol Seong, S., et al. Data Resource Profile: The National Health Information Database of the National Health Insurance Service in South Korea. *Int J Epidemiol.* **46**, 799-800 (2017).

**Supplementary Tables**

**Supplementary Table S1. Definitions of comorbidities**

| **Disease** | **Definitions** |
| --- | --- |
| **Hypertension** | At least one diagnosis (I10-13 or I15) per year and at least one claim per year for the antihypertensive medication  Or systolic/diastolic blood pressure ≥ 140/90 mmHg |
| **Diabetes mellitus** | At least one diagnosis (E10-14) per year and at least one claim per year for the prescription of antidiabetic medication  Or fasting glucose level ≥ 126 mg/dL |
| **Dyslipidemia** | At least one diagnosis (E 78) per year and at least one claim per year for the prescription of a lipid-lowering agent  or Total cholesterol ≥ 240 mg/dL |
| **CKD** | Estimated glomerular filtration rate <60 ml/min/1.73m^2^ |

Abbreviation: CKD, chronic kidney disease.

**Supplementary Table S2. Unadjusted and age, sex-adjusted analysis for the risk of incident atrial fibrillation**

| **Characteristics** | **Unadjusted HR**  **(95% CI)** | **P value** | **Age, sex adjusted HR (95% CI)** | **P value** |
| --- | --- | --- | --- | --- |
| Age (per 1 year) | 1.046 (1.046-1.047) | <0.001 | 1.047 (1.047-1.047) | <0.001 |
| <55 years | 1 (reference) |  | 1 (reference) |  |
| 55-64 years | 2.859 (2.840-2.879) | <0.001 | 1.384 (1.374-1.395) | <0.001 |
| 65-74 years | 4.840 (4.807-4.872) | <0.001 | 1.590 (1.576-1.604) | <0.001 |
| ≥75 years | 6.683 (6.616-6.751) | <0.001 | 1.425 (1.405-1.445) | <0.001 |
| Male sex | 0.955 (0.950-0.960) | <0.001 | 1.137 (1.131-1.144) | <0.001 |
| Fatty liver index (per 10) | 1.061 (1.060-1.062) | <0.001 | 1.031 (1.030-1.032) | <0.001 |
| 0-30 | 1 (reference) |  | 1 (reference) |  |
| 30-60 | 1.328 (1.320-1.337) | <0.001 | 1.097 (1.090-1.104) | <0.001 |
| ≥ 60 | 1.267 (1.257-1.278) | <0.001 | 1.188 (1.178-1.198) | <0.001 |
| BMI (per 1 kg/m^2^) | 1.035 (1.034-1.036) | <0.001 | 1.015 (1.014-1.016) | <0.001 |
| Underweight (BMI<18.5 kg/m^2^) | 0.840 (0.827-0.854) | <0.001 | 0.964 (0.949-0.980) | <0.001 |
| Normal range (18.5-23 kg/m^2^) | 1 (reference) |  | 1 (reference) |  |
| Overweight (23-25 kg/m^2^) | 1.175 (1.167-1.183) | <0.001 | 1.032 (1.025-1.039) | <0.001 |
| Obese I (25-30 kg/m^2^) | 1.264 (1.255-1.272) | <0.001 | 1.074 (1.067-1.081) | <0.001 |
| Obese II (≥30 kg/m^2^) | 1.267 (1.249-1.286) | <0.001 | 1.205 (1.188-1.223) | <0.001 |
| Waist circumference (per 1 cm) | 1.027 (1.026-1.027) | <0.001 | 1.011 (1.010-1.011) | <0.001 |
| Systolic BP (per 10 mmHg) | 1.176 (1.174-1.178) | <0.001 | 1.038 (1.036-1.040) | <0.001 |
| Diastolic BP (per 10 mmHg) | 1.153 (1.150-1.156) | <0.001 | 1.031 (1.028-1.034) | <0.001 |
| Fasting glucose (per 10 mg/dL) | 1.069 (1.068-1.070) | <0.001 | 1.027 (1.026-1.028) | <0.001 |
| <110 mg/dL | 1 (reference) |  | 1 (reference) |  |
| 110-125 mg/dL | 1.521 (1.508-1.534) | <0.001 | 1.152 (1.142-1.161) | <0.001 |
| ≥ 126 mg/dL | 1.835 (1.818-1.852) | <0.001 | 1.254 (1.243-1.266) | <0.001 |
| Total cholesterol (mg/dL) (per 10mg/dL) | 1.011 (1.010-1.011) | <0.001 | 0.999 (0.999-0.999) | <0.001 |
| GGT (mg/dL) | 1.176 (1.171-1.180) | <0.001 | 1.134 (1.129-1.139) | <0.001 |
| AST (mg/dL) | 1.468 (1.457-1.479) | <0.001 | 1.108 (1.099-1.117) | <0.001 |
| ALT (mg/dL) | 1.052 (1.047-1.058) | <0.001 | 0.981 (0.976-0.987) | <0.001 |
| Hypertension | 2.273 (2.261-2.286) | <0.001 | 1.302 (1.295-1.309) | <0.001 |
| Diabetes mellitus | 2.165 (2.148-2.182) | <0.001 | 1.276 (1.266-1.286) | <0.001 |
| Dyslipidemia | 1.478 (1.469-1.488) | <0.001 | 1.061 (1.054-1.068) | <0.001 |
| CKD (eGFR ≤60 ml/min/1.73m^2^) | 1.810 (1.794-1.827) | <0.001 | 1.061 (1.051-1.071) | <0.001 |
| Smoking status |  |  |  |  |
| Non-smoker | 1 (reference) |  | 1 (reference) |  |
| Ex-smoker | 1.147 (1.139-1.156) | <0.001 | 1.131 (1.121-1.141) | <0.001 |
| Current smoker | 0.801 (0.796-0.807) | <0.001 | 1.033 (1.024-1.041) | <0.001 |
| Alcohol consumption |  |  |  |  |
| Non-drinker | 1 (reference) |  | 1 (reference) |  |
| Mild (0-30g/day) | 0.703 (0.699-0.707) | <0.001 | 0.968 (0.962-0.974) | <0.001 |
| Regular exercise (Yes) | 0.842 (0.838-0.847) | <0.001 | 0.977 (0.972-0.982) | <0.001 |
| Low income | 1.000 (0.994-1.006) | 1.000 | 0.969 (0.963-0.975) | <0.001 |

ALT, alanine transaminase; AST, aspartate transaminase; BMI, body mass index; BP, blood pressure; CI, confidence interval; CKD, chronic kidney disease; eGFR, estimated glomerular filtration rate; GGT, gamma-glutamyl transferase; HDL, high density lipoprotein; HR, hazard ratio.

**Supplementary Table S3. Hazard ratios of the fatty liver index for incident atrial fibrillation in different BMI groups**

| **BMI** | **FLI** | **Total number** | **AF cases** | **IR*** | **Model 1**  **HR (95% CI)** | **Model 2**  **HR (95% CI)** | **Model 3**  **HR (95% CI)** |
| --- | --- | --- | --- | --- | --- | --- | --- |
| **<18.5** | 0-30 | 321,319 | 15,701 | 6.1 | 1 (reference) | 1 (reference) | 1 (reference) |
|  | 30-60 | 1,947 | 279 | 20.2 | 1.747 (1.551-1.967) | 1.615 (1.432-1.822) | 1.593 (1.412-1.797)† |
|  | ≥ 60 | 401 | 68 | 24.1 | 2.107 (1.660-2.675) | 1.926 (1.515-2.448) | 1.916 (1.507-2.436)† |
| **18.5-23** | 0-30 | 3,018,820 | 172,556 | 7.1 | 1 (reference) | 1 (reference) | 1 (reference) |
|  | 30-60 | 212,068 | 18,447 | 11.0 | 1.230 (1.211-1.250) | 1.159 (1.141-1.178) | 1.177 (1.159-1.196)† |
|  | ≥ 60 | 27,088 | 2,887 | 13.9 | 1.523 (1.468-1.581) | 1.393 (1.341-1.446) | 1.414 (1.362-1.468)† |
| **23-25** | 0-30 | 1,320,942 | 86,934 | 8.2 | 1 (reference) | 1 (reference) | 1 (reference) |
|  | 30-60 | 550,891 | 42,480 | 9.7 | 1.042 (1.029-1.054) | 1.025 (1.012-1.037) | 1.046 (1.033-1.059)† |
|  | ≥ 60 | 105,448 | 8,613 | 10.3 | 1.195 (1.168-1.223) | 1.153 (1.127-1.180) | 1.195 (1.167-1.224)† |
| **25-30** | 0-30 | 642,133 | 44,840 | 8.7 | 1 (reference) | 1 (reference) | 1 (reference) |
|  | 30-60 | 992,400 | 78,263 | 9.9 | 1.016 (1.004-1.028) | 0.984 (0.972-0.996) | 0.998 (0.986-1.011) |
|  | ≥ 60 | 589,083 | 43,475 | 9.2 | 1.092 (1.077-1.108) | 1.032 (1.017-1.047) | 1.061 (1.045-1.077)† |
| **≥ 30** | 0-30 | 6,220 | 402 | 8.0 | 1 (reference) | 1 (reference) | 1 (reference) |
|  | 30-60 | 56,818 | 4,583 | 10.1 | 1.041 (0.940-1.152) | 0.986 (0.890-1.093) | 1.000 (0.903-1.108) |
|  | ≥ 60 | 202,477 | 14,914 | 9.2 | 1.111 (1.005-1.228) | 1.021 (0.924-1.130) | 1.050 (0.950-1.162) |

*IR, incidence rate (per 1000 person-years)

†Bonferroni-corrected significance level of p <0.004 was used for multiple comparisons.

Model 1: age and sex

Model 2: Model 1 + hypertension, diabetes, dyslipidemia, chronic kidney disease, smoking, alcohol consumption, exercise, and low income

Model 3: Model 2 + systolic blood pressure, total cholesterol, fasting glucose

Abbreviation: AF, atrial fibrillation; BMI, body mass index; CI, confidence interval; FLI, fatty liver index; HR, hazard ratio; IR, incidence rate.

**Supplementary Table S4.** **Hazard ratios for incident atrial fibrillation compared to normal weight with normal FLI group as a reference group**

| **BMI** | **FLI** | **Model 2**  **HR (95% CI)** | **Model 3**  **HR (95% CI)** |
| --- | --- | --- | --- |
| **<18.5** | 0-30 | 0.985 (0.969-1.002) | 0.975 (0.960-0.992) |
|  | 30-60 | 1.751 (1.556-1.969) | 1.739 (1.546-1.966) |
|  | ≥ 60 | 2.127 (1.677-2.698) | 2.133 (1.681-2.705) |
| **18.5-23** | 0-30 | 1 (reference) | 1 (reference) |
|  | 30-60 | 1.162 (1.144-1.180) | 1.187 (1.168-1.205) |
|  | ≥ 60 | 1.404 (1.353-1.457) | 1.435 (1.383-1.489) |
| **23-25** | 0-30 | 0.999 (0.991-1.008) | 1.005 (0.996-1.013) |
|  | 30-60 | 1.035 (1.023-1.046) | 1.056 (1.044-1.067) |
|  | ≥ 60 | 1.141 (1.116-1.166) | 1.175 (1.149-1.201) |
| **25-30** | 0-30 | 1.028 (1.017-1.039) | 1.031 (1.020-1.042) |
|  | 30-60 | 1.015 (1.007-1.024) | 1.029 (1.020-1.038) |
|  | ≥ 60 | 1.067 (1.055-1.079) | 1.095 (1.083-1.107) |
| **≥ 30** | 0-30 | 1.158 (1.050-1.277) | 1.151 (1.044-1.269) |
|  | 30-60 | 1.121 (1.088-1.154) | 1.127 (1.094-1.161) |
|  | ≥ 60 | 1.108 (1.089-1.127) | 1.131 (1.112-1.150) |

Abbreviation: BMI, body mass index; CI, confidence interval; FLI, fatty liver index; HR, hazard ratio.

Model 2: age, sex, hypertension, diabetes, dyslipidemia, chronic kidney disease, smoking, alcohol consumption, exercise, and low income

Model 3: Model 2 + systolic blood pressure, total cholesterol, fasting glucose
